# Genomic analysis of primary and recurrent gliomas reveals clinical outcome related molecular features

**DOI:** 10.1038/s41598-019-52515-9

**Published:** 2019-11-05

**Authors:** Longbo Zhang, Zhiqiang Liu, Jin Li, Tianxiang Huang, Ying Wang, Lianpeng Chang, Wenjie Zheng, Yujie Ma, Fenghua Chen, Xuan Gong, Qianying Yuan, Shannon Teaw, Xinqi Fang, Tao Song, Lei Huo, Xi Li, Xuefeng Xia, Zhixiong Liu, Jun Wu

**Affiliations:** 1 Department of Neurosurgery, Xiangya Hospital, Central South University, Changsha, Hunan China; 2 Department of Neurosurgery, Yale School of Medicine, New Haven, CT United States; 3 Geneplus-Beijing, Beijing, China; 4 Department of Emergency, Xiangya Hospital, Central South University, Changsha, Hunan China; 5 Department of Pharmacology, Yale School of Medicine, New Haven, CT United States; 6 Xiangya Medical School, Central South University, Changsha, Hunan China

**Keywords:** CNS cancer, Prognostic markers

## Abstract

Tremendous efforts have been made to explore biomarkers for classification and grading on gliomas. The goal of this study was to identify more molecular features that are associated with clinical outcomes by comparing the genomic profiles of primary and recurrent gliomas and determine potential recurrence leading factors that are significantly enriched in relapse tumors. Hybrid capture based next generation sequencing (NGS) analysis was performed on 64 primary and 17 recurrent glioma biopsies. Copy number variation (CNV) was more frequent in recurrent tumors and CDKN2A/B loss was significantly enriched. In addition, overall mutations in cell cycle pathway are more common in relapse tumors. The patterns of gene sets, including IDH1/TERT and IDH1/TP53 exhibited significant difference between the groups. Survival analysis uncovered the worse disease-free survival (DFS) and overall survival (OS) associated with altered copy number and excessive activation of CELL CYCLE pathway. High Tumor Mutation Burden (TMB) was also a biomarker with great potential for poor prognosis. The assessment of genomic characteristics in primary versus recurrent gliomas aids the discovery of potential predictive biomarkers. The prognostic value of TMB in gliomas was raised for the first time.

## Introduction

Gliomas are the most frequent tumors in brain and central nervous system (CNS)^1^. According to the World Health Organization (WHO) classification, the main gliomas are subdivided by the glial cells that they originated from, including astrocytes, ependymal cells and oligodendrocytes^2^.

Since the start of The Cancer Genome Atlas (TCGA) project, there has been an increase in molecular analysis performed to explore the mutational landscape of different glioma subtypes^3–5^. The integration of genomic parameters leads to more accurate classification and grading diagnosis. Such molecular information has revealed evidence for prognosis prediction and treatment response^6–9^. For example, the existence of isocitrate dehydrogenase 1 or 2 (IDH1/2) mutation and 1p/19q deletion suggests low-grade oligodendroglioma (WHO II), while additional aberrations, including 9p and 10q loss, CDKN2A/B or RB1 deficiency and p14ARF methylation, indicates a progression to anaplastic oligodendroglioma (WHO III)^10^. Additionally, IDH1/2 mutation is associated with prolonged PFS and OS and higher response rate to temozolomide (TMZ)^8^, and 1p/19q codeletion is a biomarker to predict better response to combined radiotherapy and chemotherapy^11^. Because of this, the majority of the molecular studies thus far are focused on exploring the genomic landscape, patient stratification or response prediction in certain glioma subtype. Recently, several studies have focused on tumor evolution by comparing the genomic landscape of paired primary and recurrent samples^12–15^. However, the population size of these studies was small and the knowledge of driver events for glioma recurrence are still limited.

We noticed that the use of immune checkpoint inhibition has contributed to significant clinical benefits in the treatment of several cancers, including melanoma^16,17^, non-small cell lung cancer (NSCLC)^18^, but not in glioma^19^. The first large randomized clinical trial of PD-1 in the setting of GBM, CheckMate 143, showed a failure of nivolumab to prolong overall survival of patients. TMB has been shown as a biomarker of response to immunotherapy in recent studies^20^, and beyond that, is associated with better prognosis in resected NSCLC patients^21^. The distribution of TMB has been described in previous studies, and reported to be increased after treatment of TMZ^22^. However, the relationship between TMB and clinical outcomes in glioma is still unclear.

In this study, we retrospectively analyzed the genomic alteration of 81 resected glioma samples from distinct pathological subtypes. All specimens were sequenced by using a targeted panel of 1021 cancer related genes. By comparing molecular features of primary and recurrent gliomas, some characteristics were notably enriched in recurrent group but relatively rare in primary tumors, including the occurrence of CNV, co-occurance of IDH1 and TERT, inactivated cell cycle signaling pathway and low TMB, which may provide clinical insights on tumor relapse and poor prognosis.

## Results

### Characteristics of patient cohort

A total of 81 glioma specimens from 80 patients were collected in this study, including 64 primary and 17 recurrent surgical resected tumors, two of which were matched. The clinical characteristics of samples were summarized in Table 1. It is worth noting that despite different clinical features, the patient distribution was similar between primary and recurrent gliomas, thus allowing us to better evaluate and compare the genomic features between the groups.

**Table 1:** Clinical features of patients with primary and recurrent glioma.

Features	All (%) n = 81	Primary (%) n = 64	Recurrent (%) n = 17	p value
**Sex**				
Male	49 (60.49)	40 (62.5)	9 (52.94)	0.66
Female	32 (39.51)	24 (37.5)	8 (47.06)	
**Age**				
<=40	25 (30.86)	22 (34.38)	3 (17.65)	0.28
>40	54 (66.67)	41 (64.06)	13 (76.47)	
Unknown	2 (2.47)	1 (1.56)	1 (5.88)	
**WHO**				
I	2 (2.47)	2 (3.13)	0 (0)	0.34
II	28 (34.57)	23 (35.94)	5 (29.41)	
I	19 (23.46)	15 (23.44)	4 (23.53)	
IV	31 (38.27)	24 (37.50)	7 (41.18)	
Unknown	1 (1.23)	0 (0)	1 (5.88)	
**Glial cell**				
GBM	26 (32.10)	20 (31.25)	6 (35.29)	0.84
Astrocyte	42 (51.85)	34 (53.13)	8 (47.06)	
Oligodendrocyte	10 (12.35)	8 (12.35)	2 (11.769)	
Ependymal cell	1 (1.23)	1 (1.23)	0 (0)	
Unknown	2 (2.47)	1 (1.23)	1 (5.88)	
**MGMT methylation**				
+	30 (37.04)	23 (28.40)	7 (41.18)	0.69
−	31 (38.27)	26 (32.10)	5 (29.41)	
NA	20 (24.69)	15 (18.52)	5 (29.41)	

### Somatic variants

The genomic analysis was performed using hybrid capture based targeted sequencing. Overall, about 870 of mean tumor target coverage was achieved, with 99.7% of bases over 50-fold coverage.

**Figure 1:**
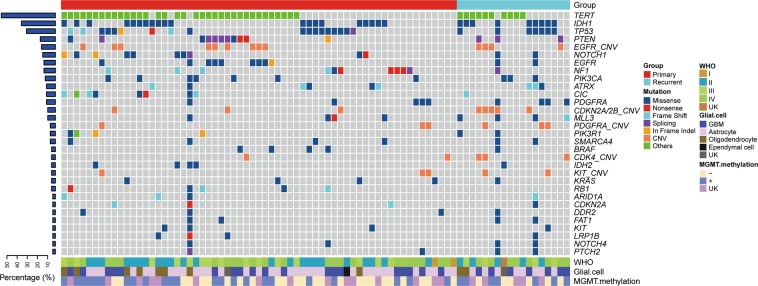
Mutational landscape of primary and recurrent gliomas. Each column represents individual patients, and mutated genes are listed on the y-axis. Different colors refer to mutational functions and clinical information as indicated.

A total of 840 nonsynonymous somatic mutations from 301 genes were identified. As previously reported, the most mutated genes in glioma were TERT, IDH1, TP53, PTEN, NOTCH1 and EGFR (Fig. 1). In particular, all IDH1 mutations were found to substitute Arginine residue at codon 132, of which 93% were R132H (27/29). In contrast, R132G and R132C substitutions were rare and identified only once. Missense and indel variants in PTEN, NOTCH1 and EGFR were found to be scattered throughout the genes (Supplementary Fig S1B). Regarding copy number variants, amplification of EGFR, PDGFRA, CDK4, KIT and loss of CDKN2A/2B were the most common copy number aberrations. Additionally, structural variants of genes were also evaluated. FGFR3/TACCA3 and EGFR/EGFR rearrangements were detected in 3 and 2 tumors respectively. Interestingly, synchronous amplification was observed in 1/3 FGFR3 and 2/2 EGFR tumors, suggesting the strong association between CNV and SV variants in gliomas as previous described^3^.

Furthermore, we analyzed 303 GBM and 509 LGG samples from TCGA database and similar mutation spectrum was observed (Supplementary Fig S1A). This demonstrated a suitable panel for genomic studies on gliomas. When we compared the most mutated 15 genes in this study with TCGA and MSK database, we did not find tremendous differences, but higher mutational frequency of NOTCH1, PDGFRA and MLL3, and lower mutational frequency of ATRX were shown in this study.

### Concurrent and exclusive gene sets

**Figure 2:**
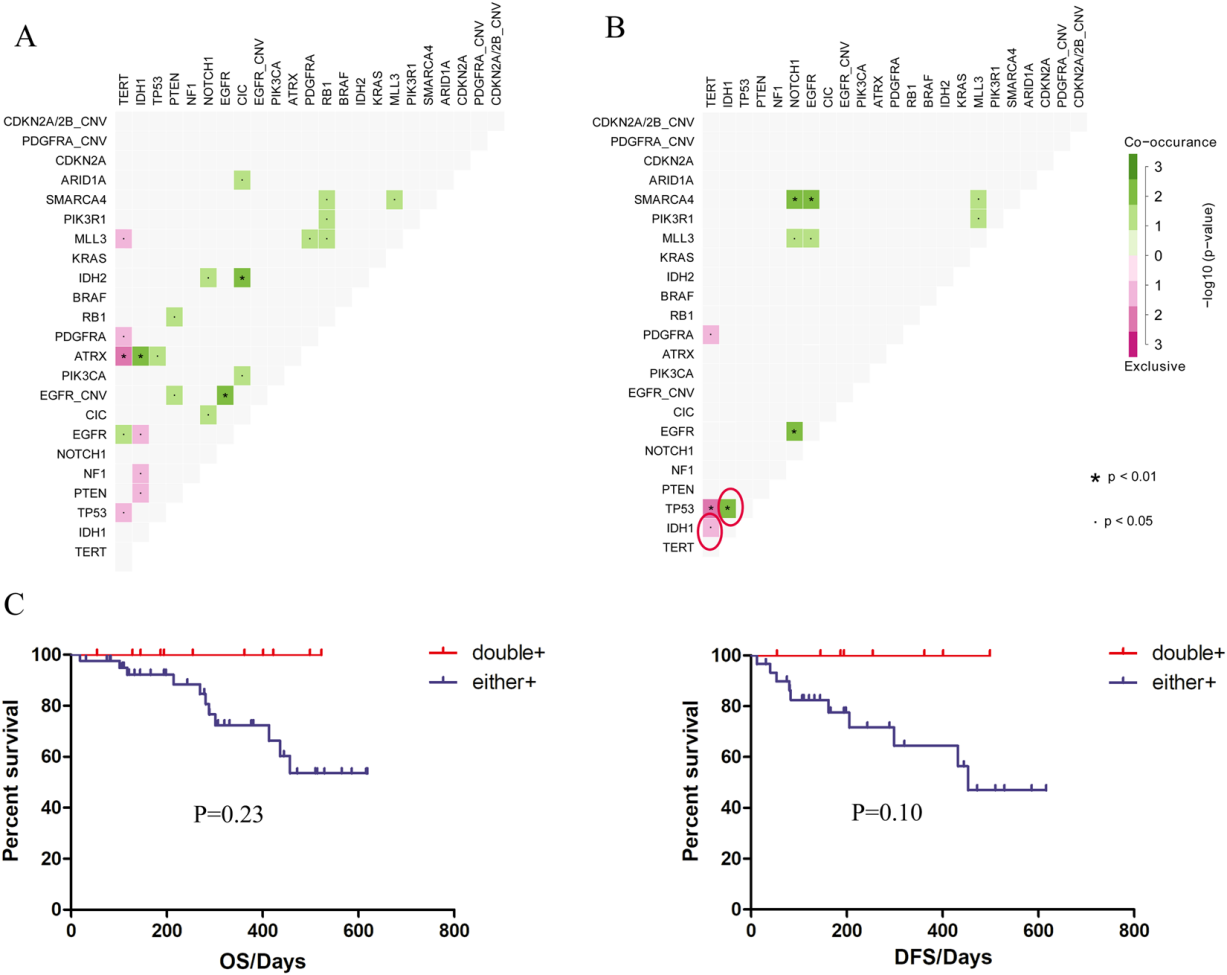
Effects of somatic interactions on DFS and OS in primary subset. (**A**,**B**) Significant exclusive or co-occurance gene sets with Fisher’s Exact test are indicated in primary (**A**) and recurrent (**B**) tumors. (**C**) Survival analysis of TERT/IDH1 gene pattern in primary glioma patients. P values were calculated using the Log-rank Test.

Genomic landscape studies have uncovered some co-occurring or mutually exclusive mutations in different cancer types. For instance, EGFR mutated exclusively with other known oncogenic drivers like KRAS, ROS1, MET and ALK aberrations in NSCLC^23^. Concurrent PDGFRA and EGFR alterations, exclusive of EGFR amplification and IDH1 mutation were described in GBM and low-grade gliomas respectively^24^. However, what patterns of gene pairs in recurrent gliomas and whether the patterns would influence the patient outcome are still unknown. Here, we investigated the mutual exclusivity and cooccurrence of mutations in genes mutated in at least 5 samples (Supplementary Fig. S2). The most notable exclusive gene sets were TERT and ATRX, TERT and PDGFRA and TERT and TP53. More concurrent aberration sets were identified including IDH1 and ATRX. Interestingly, when looking at the somatic interactions in primary and recurrent gliomas, there were some co-occurrence and mutual exclusivity of mutations only existing in the recurrent subset, including TERT and IDH1 (p = 0.050), IDH1 and TP53 (p = 0.002) (Fig. 2A,B). We further investigated whether the patterns of those gene sets were associated with prognosis in primary gliomas. As hypothesized, the exclusivity of TERT and IDH1 contributed to poorer DFS and OS (Fig. 2C) but the differences were not statistically significant. The concurrence of IDH1 and TP53 did not exhibit different DFS or OS (Supplementary Fig. S2).

### CNV

**Figure 3:**
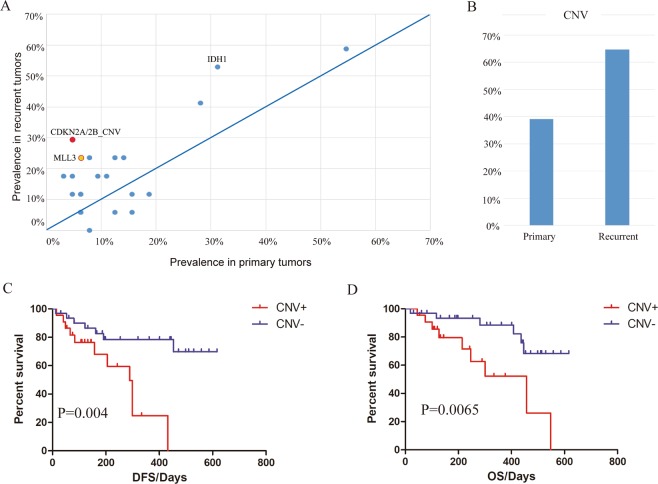
Comparison of SNVs or CNVs between primary and recurrent gliomas. (**A**) The prevalence of genes mutated in at least 5 samples across all tumors were calculated in different subsets. Red and yellow dots represent p < 0.05 and p < 0.1 respectively (Fisher’s Exact). (**B**) The incidence of CNV in primary and recurrent tumors. (**C**,**D**) Impact of CNV status on DFS (**C**) and OS (**D**). P values were calculated using the Log-rank Test.

Next, we examined the proportion of recurrent somatic aberrations within the groups to differentiate mutational patterns between primary and recurrent tumors (Fig. 3A). We found that CDKN2A/2B loss^25^, which has been described as an indicator of poor survival in astrocytoma, was significantly enriched in recurrent cohort. Moreover, the incidence of CDK4 (18% vs. 3%), somatic mutations of MLL3 (24% vs. 6%), PDGFRA (18% vs. 5%) and IDH1 (53% vs. 31%) were more frequent in recurrent patients, which are likely to be linked to poor survival. Additionally, recurrent gliomas, as well as high grade gliomas, featured more frequent copy number variants (65% recurrent versus 39% primary tumors, 60% high grade versus 20% in low grade tumors) (Fig. 3B, Supplementary Fig. S3), implicating the strong association of CNV with patient’s survival. Indeed, for primary tumors, patients carrying CNV exhibited a significantly inferior DFS (HR = 4.59, 95% CI = 1.63–12.93) and OS (HR = 4.89, 95% CI = 1.56–15.35) rate (Fig. 3C).

### Signaling pathway

**Figure 4:**
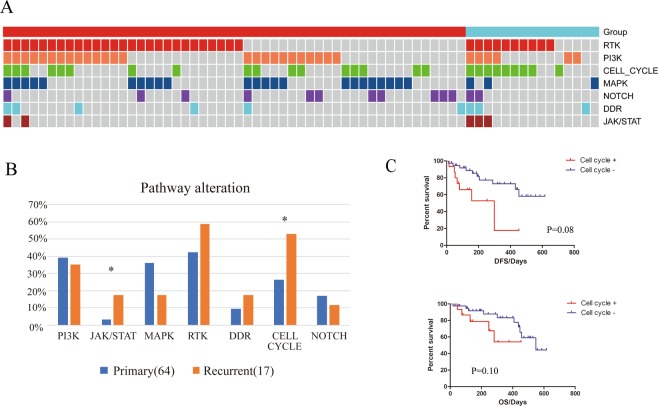
Analysis of key gene alterations grouped by biological function. (**A**) The landscape of signaling pathway alterations in glioma tumors. (**B**) Prevalence comparison of altered pathways in primary and recurrent subset with Fisher’s Exact test. (**C**) Survival analysis of primary patients with Cell cycle alterations versus patients without. P values were calculated using the Log-rank Test.

From the analysis above, we observed that several cell cycle related genes, such as CDKN2A/2B (14% vs. 3%), CDK4 (10% vs. 0) and RB1(10% vs. 0), were dominant in recurrent gliomas as compared to primary gliomas, so we further clustered the mutated genes according to their functional characteristics. Pathway constituents are defined in the Supplemental table S1 based on previous studies^26^. RTK, PI3K, CELL CYCLE, MAPK, JAK/STAT, NOTCH and DDR signaling pathway were investigated. Overall, at least one RTK alteration was found in nearly half (45.7%, 37/81) of gliomas and PI3K mutations were detected in 38.3% (31/81) of all samples. 26/81 (32.1%) have at least one SNV or CNV in the cell cycle regulation pathway (Fig. 4A). No significant concurrent or exclusive pathway sets were observed. When comparing the proportion of altered pathways between primary and recurrent gliomas, we found that dysregulated cell cycle (53% vs. 27%) and JAK/STAT pathway (18% vs. 3%) was significantly more abundant in the recurrent cohort (Fig. 4B). In contrast, MAPK pathway showed higher mutation frequency in primary gliomas, though not statistically significant. In order to figure out whether recurrent enriched pathway alterations contribute to higher risk of relapse or worse prognosis, survival analysis was performed. Consistent with the hypothesis, patients harboring cell cycle dysregulation showed significantly shorter DFS compared to the unchanged group. A similar tendency was observed in the OSanalysis, though not statistically significant.

### TMB

**Figure 5:**
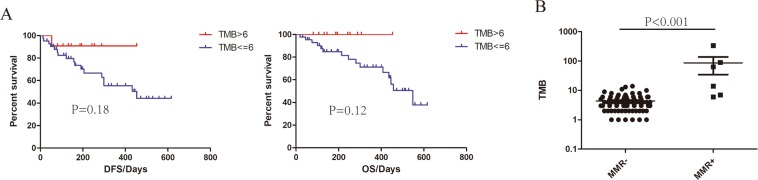
Impact of tumor mutation burden on prognosis. (**A**) Effect of TMB on DFS and OS. P values were calculated using the Log-rank Test. (**B**) The comparison of TMB between groups with and without mutated MMR genes.

At present, TMB is recognized not only as one of the primary biomarkers for immunotherapy, but is also associated with prognosis in resected NSCLC patients^21^. In order to explore its role in patients with resected glioma, nonsynonymous mutations were calculated. The median TMB among all samples was 4 mutations per megabase (Mb). When tumors from the primary group were categorized at 25% quantiles of TMB, 6, patients with higher TMB showed more favorable outcomes (HR = 0.43, 95% CI = 0.12–1.48 for DFS, HR = 0.30, 95% CI = 0.06–1.36 for OS). A potential reason for insignificant difference may be insufficient follow-up time (Fig. 5A). Next, we questioned what causes could contribute to high TMB. Previously, TMZ has been reported to result in higher mutation burden^22^, and this was confirmed in our study since higher TMB was shown in recurrent patients, whom had been treated with TMZ, as compared to those with primary gliomas (Supplementary Fig S4). Moreover, mutations in mismatch repair (MMR) genes often showed higher TMB (median TMB = 14) than MMR negative tumors (Fig. 5B), which is consistent with previous studies. Overall, our findings suggest that TMB would serve as a potential marker for primary resected gliomas.

## Discussion

To our knowledge, extensive efforts have been made to characterize distinct molecular subgroups in glioma, and this has enabled the stratification of patients with better or worse prognosis. In order to determine the driver events for glioma recurrence, we performed comprehensive analyses and comparison between primary and recurrent gliomas by using the NGS data from primary and recurrent glioma samples. Upon analysis of these results, we provided valuable evidence regarding the role of CNV, the pattern of IDH1 with TERT, cell cycle signaling pathway and TMB level as potential prognostic biomarkers.

The mutational prevalence of some genes was different between this study and TCGA/MSK cohort. The reason might be the different human lineages within, or the divergent clinical features of the three cohorts. Further analysis based on larger Chinese cohort would help to explore the genomic landscape comparison.

CDKN2A/B loss was found to be significantly enriched in recurrent gliomas. We were unable to perform survival analysis since only two primary gliomas harbored this alteration. However, previous studies have illustrated the relationship between CDKN2A/2B loss and poor outcomes, in terms of overall survival^25^. This indicates that identifying risk stratification factors from recurrent enriched features is reasonable and feasible.

From the oncoprint output, we noticed higher frequency of CNV events in recurrent gliomas than primary tumors. Further analysis displayed a strong association between CNV presence and shorter DFS and OS. It is clear that DNA copy number alterations, especially amplification on chromosome 7 (including EGFR/MET/CDK6) and chromosome 4 (including PDGFRA) and loss on chromosome 9 (including CDKN2A/B), are common in gliomas^3,5^. It has been reported that CNV pattern in IDH mutated gliomas is distinct from IDH wildtype group, which exhibited poorer prognosis^4,5,27^. Even though we did not observe significantly higher frequency of EGFR gain and PTEN loss in IDH unchanged samples as previously described^28^, IDH intact tumors demonstrated notably higher overall copy number changes compared to IDH1 mutated tumors (p = 0.012, Fisher’s exact test). This demonstrated the effect of CNV, the degree of which reflects the chromosomal stability, on DFS and OS might be associated with the IDH status. Moreover, it is worth noting that different panels with distinct covered regions would impact this result.

To integrate the genetic alterations, including SNV, CNV and SV, altered genes were mapped onto major biological pathways. RTK/RAS/PI3K (88%), P53 (87%) and RB (78%) signaling were three core pathways reported by TCGA study in GBM. Recently, Ellis et. al reported the altered pathway frequency in primary and recurrent GBM^29^. Their data showed a discordant comparison, RB cell cycle pathway is more prevalent in primary GBM than in recurrent GBM (19% vs. 0%). The difference may be due to insufficient recurrent GBM (n = 8) in their study, or different cluster criterion for gene grouping. Likewise, it has been suggested that alterations in this pathway are more frequent in higher-grade (Grades III and IV) gliomas, but the incidence in different grade tumors are similar. Therefore, the shorter DFS and OS by cell cycle pathway activation in this study is due to excessive cell proliferation, rather than higher grading.

Another kind of potential predictive biomarker we recognized is somatic interaction of gene pairs. Even though the association of gene sets pattern (IDH1/TERT) and prognosis is not statistically significant in this study, a similar hypothesis has been described in other studies. Previous studies have mentioned that the patterns of IDH1/2 and TERT were involved in glioma classification. Patients with both mutations in TERT promoter IDH1/2 were found to have had the best OS^6,7^.

Although immune checkpoint inhibitor tremendously improved overall survival for patients with diverse solid tumor types, there is no promising data from current clinical trials for the treatment of gliomas thus far^30^. Many studies have demonstrated the infiltration of CD8 positive cells in GBM is normally weaker than that in tumors like melanoma and lung cancer^31^. Apart from the impact of immune microenviroment, intrinsically weak PD-L1 expression level in GBM may be another reason^32^. As a genomic biomarker of response to immunotherapy, TMB was also investigated in this study. It is known that the use of TMZ would induce high level of TMB, which could explain the higher TMB we observed in recurrent gliomas. Additionally, patients with MMR gene mutations correspond to higher TMB in gliomas. However, the predictive effect on DFS or OS in gliomas have yet to be studied. In this study, we found a clear trend toward better prognosis with increasing TMB in patients. Furthermore, a recent study, irrelevant to immunotherapy, has implicated that high TMB leads to better prognosis in NSCLC^20^. The insignificance may be due to insufficient follow-up time.

There are several limitations in this study. It is important to note that the study cohort includes patients with varied clinical features, including varying pathology and grading. This gives rise to small sample sizes in each subgroup. Furthermore, paired data in this study is limited, and since matched primary and recurrent gliomas are often imperfect, more robust conclusions may only be drawn from paired data. In addition, short follow-up time weakens survival analysis in some aspects and all sequences and data analysis conducted in this study were panel based, thus variation may exist by using different panels.

Taken together, identifying genomic characteristics in recurrent gliomas at individual gene, gene sets, variation type and pathway dimensions aids in discovering potential prognostic biomarkers for glioma patients. In this study, we raised the interesting finding that TMB is a potential stratification marker for clinical outcome.

## Material and Methods

### Subjects and measurements

81 surgical gliomas from the Department of Neurosurgery, Xiangya Hospital, Central South University were collected in this study. The histological subtypes were identified on the basis of the morphologic characteristics of the tumor and the results of IHC according to the WHO 2016 classification criteria of gliomas. Clinical information was extracted from medical records, including age, sex and diagnostic related information. All patients signed an informed consent. This study was approved by Medical Ethics Committee of Xiangya Hospital, Central South University (No. 201612800) and all methods were performed in accordance with ethical regulations.

### Comprehensive genomic profiling

Genomic DNA (gDNA) from gliomas was isolated by using DNeasy Tissue Kit (Qiagen, Hilden, Germany), according to the manufacturer’s protocol. A 1021 gene panel with potential clinical relevance was used to capture target regions. DNA sequencing was carried out with paired-end reads on the Illumina HiSeq sequencing system.

### Somatic calling

MuTect (version 1.4) and NChot^33^ were used for single nucleotide variants (SNV) calling. Small insertions and deletions (indels) were called by GATK. For somatic copy-number alteration, CONTRA (v2.0.8) was performed. An in-house algorithm was used to identify split-read and discordant read-pair to identify SVs. All candidate variants were manually verified with the integrative genomics viewer browser.

### Statistical analysis

Fisher’s exact test or Chi-square test was used for comparison of categorical variables. DFS and OS was analyzed by the Kaplan-Meier plots and the difference between groups was evaluated by log-rank test. All statistical analyses were performed with SPSS (v. 21.0) or GraphPad Prism (v. 6.0) software. Statistical significance was defined as a two-sided P-value < 0.05.

### Ethical approval and informed consent

Informed consent was obtained from all participants or their legal guardians. This study was approved by Medical Ethics Committee of Xiangya Hospital, Central South University (No. 201612800) and all methods were performed in accordance with ethical regulations.

## Supplementary information

Supplementary Information

## Data Availability

The datasets used and/or analysed during the current study are available from the corresponding author upon reasonable request.
