# Supplementary material for: Genomic analysis of primary and recurrent gliomas reveals clinical outcome related molecular features
Source: Sci Rep. 2019 Nov 5;9:16058. doi: 10.1038/s41598-019-52515-9 (PMC6831607; doi:10.1038/s41598-019-52515-9)
Supplement: Supplementary file 1 — Supplementary Information [file 41598_2019_52515_MOESM1_ESM.docx]

**Genomic analysis of primary and recurrent gliomas reveals clinical outcome related molecular features**

Longbo Zhang^1,2 #^, Zhiqiang Liu^2 #^, Jin Li^3 #^, Tianxiang Huang^2^, Ying Wang^4^，Lianpeng Chang^3^, Wenjie Zheng^3^, Yujie Ma^2^, Fenghua Chen^2^, Xuan Gong^2^, Qianying Yuan^5^, [Shannon Teaw](https://onlinelibrary.wiley.com/action/doSearch?ContribAuthorStored=Teaw%2C+Shannon)^1^, Xinqi Fang^6^, Tao Song^2^, Lei Huo^2^, Xi Li^2^, Xuefeng Xia^3^, Zhixiong Liu^2^, Jun Wu^2^*

^1^Department of Neurosurgery, Yale School of Medicine, New Haven, CT

^2^Department of Neurosurgery, Xiangya Hospital Central South University. Changsha, Hunan, China

^3^Geneplus-Beijing, China

^4^Department of Emergency, Xiangya Hospital Central South University. Changsha, Hunan, China

^5^Department of Pharmacology, Yale School of Medicine, New Haven CT

^6^Xiangya Medical School, Central South University. Changsha, Hunan, China

^#^ These authors contributed equally to this work

* Corresponding author

**Correspondence to:** Jun Wu, Department of Neurosurgery, Xiangya Hospital Central South University, No.87 Xiangya Road, Kaifu District, Changsha, Hunan, 41008, China.

Tel: +86- 0731- 89753039;

Fax: +86-0731- 89753039;

Email: [j](mailto:j)ianjuncs@foxmail.com

| **Table S1**. Genes considered in pathway analyses. | | | | | | |
| --- | --- | --- | --- | --- | --- | --- |
| **PI3K** | **JAK/STAT** | **MAPK** | **RTK** | **DDR** | **cellCycle** | **NOTCH** |
| *PIK3CA* | *JAK1* | *RAF1* | *EGFR* | *RAD50* | *RB1* | *NOTCH1* |
| *PIK3C2B* | *JAK2* | *BRAF* | *FGFR1* | *RAD51* | *CCNE1* | *NOTCH2* |
| *PIK3CB* | *JAK3* | *HRAS* | *FGFR3* | *RAD51D* | *CCND1* | *NOTCH3* |
| *PIK3CG* | *STAT3* | *KRAS* | *FGFR4* | *BRCA1* | *CDK4* | *NOTCH4* |
| *PIK3R1* |  | *NRAS* | *ERBB2* | *BRCA2* | *CDK6* |  |
| *PIK3R2* |  | *SHC1* | *ERBB3* | *MLH1* | *CDK8* |  |
| *AKT1* |  | *SHC2* | *ERBB4* | *MLH3* | *CDK12* |  |
| *AKT2* |  | *MAPK3* | *IGF1R* | *MSH2* | *CDK13* |  |
| *AKT3* |  | *MAPK1* | *IGF2R* | *MSH3* | *CCND2* |  |
| *MTOR* |  | *MAP3K1* | *EPHA1* | *MSH6* | *CDKN2A* |  |
| *PTEN* |  | *MAP2K1* | *EPHA2* | *ATM* | *CDKN2B* |  |
| *PDK1* |  | *MAP3K13* | *EPHA3* | *ATR* | *CDKN2C* |  |
| *TSC1* |  | *NF1* | *EPHA5* |  | *CDKN1B* |  |
| *TSC2* |  | *NF2* | *DDR2* |  |  |  |
| *RICTOR* |  |  | *DDR5* |  |  |  |
|  |  |  | *MET* |  |  |  |
|  |  |  | *PDGFRA* |  |  |  |
|  |  |  | *PDGFRB* |  |  |  |

**Supplementary Figures**

**Supplementary figure S1.** Mutational landscape. (A) Gene mutation frequency in TCGA database. (B) Distribution and location of amino acid changes in the indicated genes. Colored boxes indicate functional domains of the sequence. (C) The mutation frequency comparison between this study and TCGA.


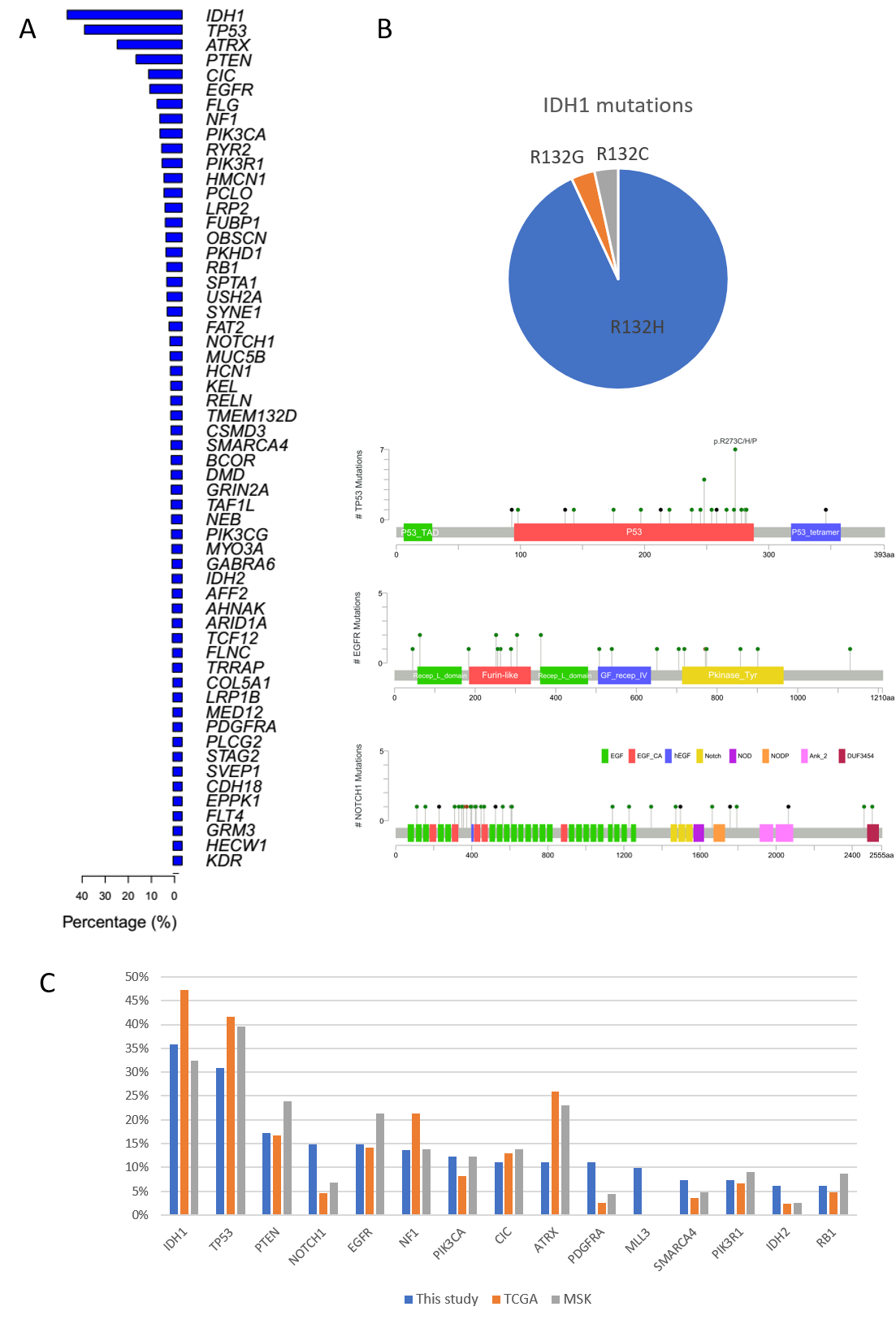


**Supplementary figure S2.** Somatic interactions in all glioma tumors. (A) Somatic interactions in all glioma tumors. (B) Survival analysis of IDH1/TP53 gene pattern in primary glioma patients. *P* values were calculated using the Log-rank (Mantel-Cox) Test.


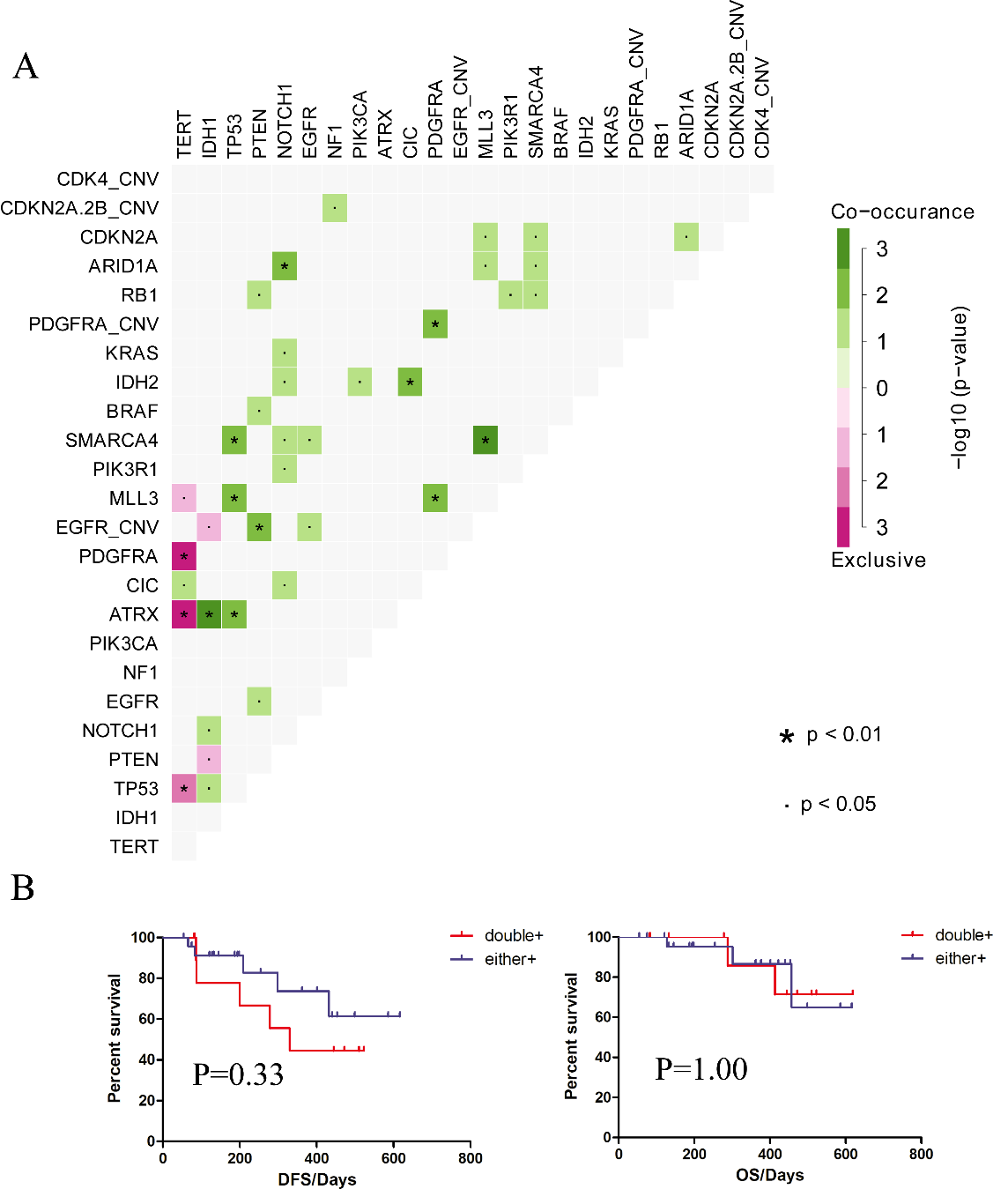


**Supplementary figure S3.** Mutational differences between Low-grade and High-grade gliomas according to the WHO guidelines. *, statistically significant differences between the groups with Fisher’s Exact Test. *, p<0.05, **, p<0.01, ***, p<0.001.


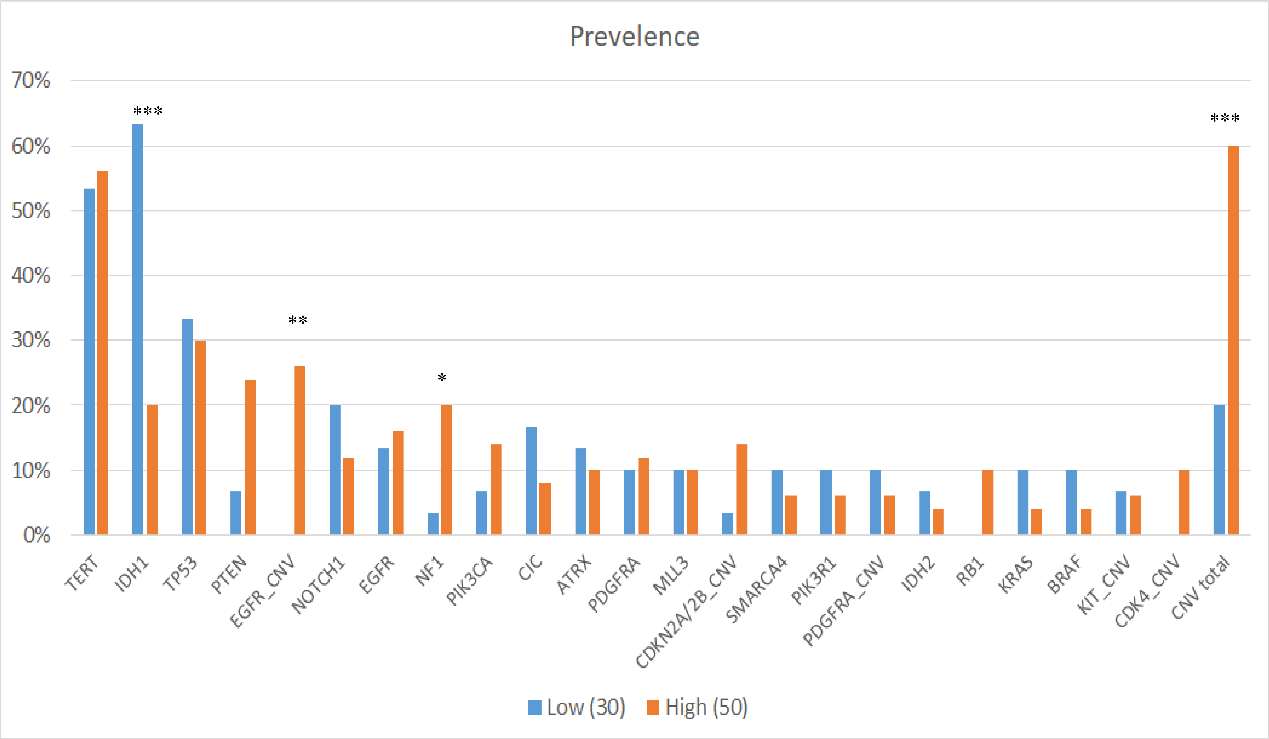


**Supplementary figure S4.** Comparison of TMB in primary and recurrent group.


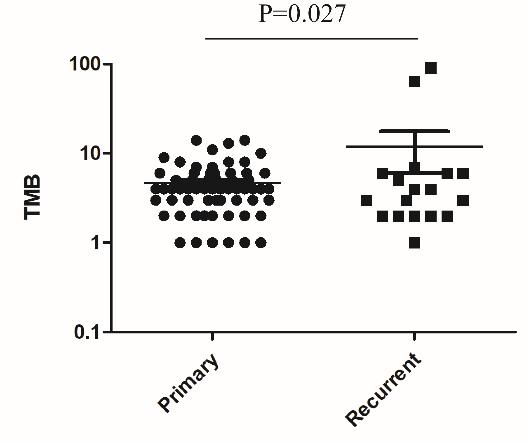


**Supplementary figure S5.** The association between IDH1 mutation and CNV status.
